# Altered fractionation short-course radiotherapy for stage II-III rectal cancer: a retrospective study

**DOI:** 10.1186/s13014-020-01566-8

**Published:** 2020-05-14

**Authors:** Hans Geinitz, Carsten Nieder, Lukas Kocik, Christine Track, Johann Feichtinger, Theresa Weingartner, Kurt Spiegl, Barbara Füreder-Kitzmüller, Johanna Kaufmann, Dietmar H. Seewald, Reinhold Függer, Andreas Shamiyeh, Andreas L. Petzer, David Kiesl, Josef Hammer

**Affiliations:** 1 Department of Radiation Oncology, Ordensklinikum Linz Barmherzige Schwestern, Seilerstätte 4, 4010 Linz, Austria; 2 Department of Oncology and Palliative Medicine, Nordland Hospital, Bodø, Norway; 3 Department of Clinical Medicine, UiT - The Arctic University of Norway, Tromsø, Norway; 4 Department of Radiotherapy, Oberoesterreichische Gesundheitsholding GmbH, Salzkammergut Klinikum Vöcklabruck, Vöcklabruck, Austria; 5 Deptartment of Surgery, Ordensklinikum Linz Barmherzige Schwestern – Elisabethinen, Linz, Austria; 6 Department of Surgery, Kepler Universitaetsklinikum, Linz, Austria; 7 Department of Internal Medicine I for Hematology with Stem Cell Transplantation, Hemostaseology and Medical Oncology, Ordensklinikum Linz Barmherzige Schwestern – Elisabethinen, Linz, Austria; 8 Department of Internal Medicine - Hematology and Oncology, Kepler Universitaetsklinikum, Linz, Austria

**Keywords:** Stage II-III rectal cancer, Neoadjuvant altered fractionation short-course radiotherapy, Low anterior resection syndrome (LARS)

## Abstract

**Purpose:**

To report the long-term outcomes of neoadjuvant altered fractionation short-course radiotherapy in 271 consecutive patients with stage II-III rectal cancer.

Patients and Methods: This was a retrospective single institution study with median follow-up of 101 months (8.4 years). Patients who were alive at the time of analysis in 2018 were contacted to obtain functional outcome data (phone interview). Radiotherapy consisted of 25 Gy in 10 fractions of 2.5 Gy administered twice daily. Median time interval to surgery was 5 days.

**Results:**

Local relapse was observed in 12 patients (4.4%) after a median of 28 months. Overall survival after 5 and 10 years was 73 and 55.5%, respectively (corresponding disease-free survival 65.5 and 51%). Of all patients without permanent stoma, 79% reported no low anterior resection syndrome (LARS; 0–20 points), 9% reported LARS with 21–29 points and 12% serious LARS (30–42 points).

**Conclusion:**

The present radiotherapy regimen was feasible and resulted in low rates of local relapse. Most patients reported good functional outcomes.

## Introduction

Pelvic radiotherapy is playing an important role in the treatment of rectal cancer and has, from a historical point of view, improved disease control already in the era that preceded effective resection strategies [1, 2]. Many randomized trials have proven that even with now standard total mesorectal excision (TME) surgery, preoperative radiotherapy [3] or chemoradiotherapy [4, 5] improve local control. Radiotherapy is mainly given in cases where tumors are located in the distal and middle rectum, and preoperative schedules have proven to be more efficient and better tolerated than postoperative ones [4]. Locally advanced rectal cancer (LARC) is usually classified as T3, T4 or anterior distal T2, where the risk of local recurrence is significant with surgery alone. Despite increasing availability of prospective randomized studies, there is still room for differing treatment strategies, e.g. in different countries [6]. Commonly, centers preferring short-course neoadjuvant radiotherapy offer the classical 25 Gy in 5 fractions of 5 Gy regimen [7, 8]. However, an Austrian group has introduced an altered fractionation variant (25 Gy in 10 fractions of 2.5 Gy administered twice daily) that was hypothesized to improve tolerability [9], in particular the development of lower anterior resection syndrome (LARS) [10]. The latter schedule has also been adopted in Linz, Austria, in 2002 and the purpose of the present study is to report the long-term outcomes in 271 consecutive patients.

## Patients and methods

**Fig. 1:**
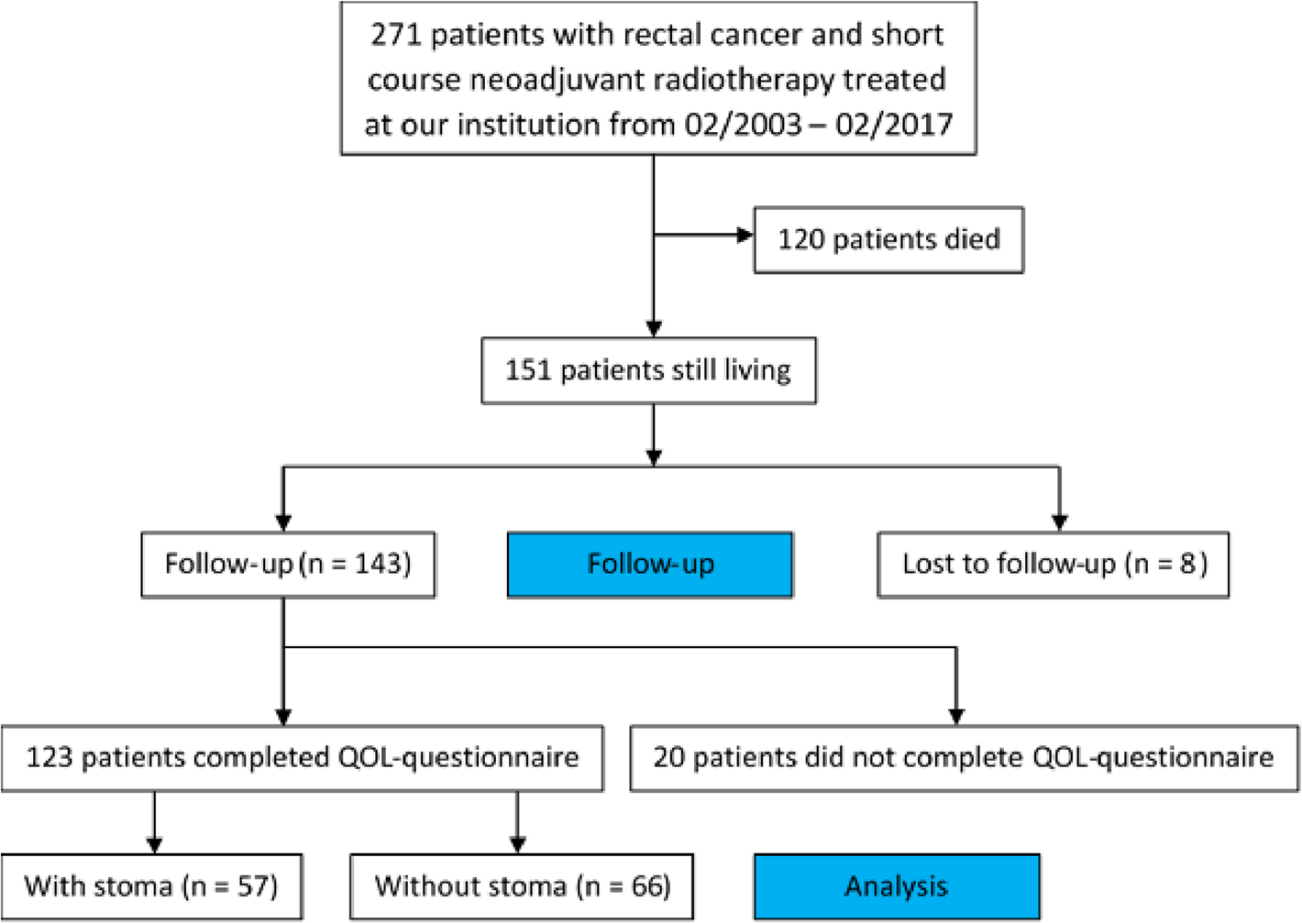
CONSORT Diagram

We performed a retrospective single institution cohort study with long-term follow-up. The study was approved by the local ethics committee. The inclusion period was 2002–2017. The median follow-up of the 271 patients was 101 months. Patients who were alive at the time of analysis in 2018 were contacted to obtain standardized functional outcome data (phone interview; Fig. 1). Date and patterns of relapse were abstracted from the hospital’s patient records, with local relapse, disease-free survival and functional outcome (LARS) as co-primary endpoints. Overall survival was assessed, too. If no event of interest had occurred, patients were censored at the time of last documented contact with the hospital.

To outline the principles of multidisciplinary assessment and therapy, patients with histologically proven adenocarcinoma of the rectum without evidence of distant metastases were eligible for neoadjuvant short-course radiotherapy, if transmural extension was to be expected upon digital examination, rectoscopy, endosonography, pelvic computed tomography (CT) and magnetic resonance imaging (MRI) scans. A complete resection of all visible disease extent, either by low anterior resection (LAR) with primary anastomosis or by abdominoperineal resection (APR), was expected feasible by the tumor board members. Selected patients not included in this study received long-term preoperative chemoradiotherapy with downsizing intent. All patients received flexible and rigid endoscopy, a CT of the abdomen and pelvis and a chest x-ray for staging (in later years thorax CT). One hundred and fifty-five of 271 patients (56%) additionally obtained an MRI of the pelvis. The percentage of patients staged with MRI increased over time: from 39% in the early years (2002–2006) to 82% at the end of the study period (2011–2017). High-risk features such as threatened radial margin or extramural vascular invasion (EMVI) were not regularly assessed.

The clinical target volume included the primary tumor, the mesorectal tissue including perirectal and presacral nodes, and internal iliac lymph nodes. The caudal boundary of the clinical target volume was at 5 cm caudal to the macroscopic tumor. Thus, the anus was included only in very low tumors. The perineum was not included in the target volume even if an APR was planned. Patients were treated in prone position (belly board) with comfortably full bladder, typically with conformal three field techniques, with a posterior and two lateral opposing wedged fields. Dose per fraction was 2.5 Gy calculated at the ICRU (International Commission on Radiation Units and Measurements) reference point. Two fractions with intervals between fractions of at least 6 h were delivered every day (25 Gy within 1 week). Surgery was planned within 1 week after radiotherapy (actual median 5 days, range 3–43 days). Depending on the location and extent of the carcinoma low anterior rectal resection or abdominoperineal amputation combined with TME were performed. None of the patients received neoadjuvant chemotherapy. Of all 271 patients, 164 received no adjuvant chemotherapy (61%), 62 received adjuvant monotherapy (23%, 5-FU or capecitabine) and 31 were treated with adjuvant polychemotherapy (11%). No information on adjuvant chemotherapy was available for 14 patients (5%).

Follow-up was every 3 months during the first year post-treatment, every 6 months during the second and third years, and yearly thereafter. Within the first 3 years, patients received an endoscopy (rectoscopy or recto-sigmoidoscopy) every 6 months and yearly afterwards. We scheduled a total colonoscopy after 1 year. During the first year, a biannual CT of the abdomen and pelvis was employed and yearly thereafter. During the first 8 years of the study period, we used an ultrasound of the liver at approximately every second follow up visit instead of an abdominal CT scan. At each visit, laboratory-tests were taken including carcino-embryonic antigen (CEA) and liver enzymes. During the first study years, patients received a yearly chest X-ray, after 2010 this was abandoned and substituted by a thorax CT in case of symptoms or a rising CEA.

Bowel function was assessed by telephone by directly inquiring the following standardized LARS items for patients without permanent stoma ([11]; Appendix 1): (1) Uncontrollable flatulences. (2) Accidental leakage of liquid stool. (3) Defecation frequency. (4) Repeated defecation within 1 hour. (5) Strong urgency to open the bowels. Patients with stoma answered the respective items from the EORTC QLQ CR 29 quality of life questionnaire ([12]; Appendix 2). Furthermore, the following symptoms were inquired and graded from 0 (no symptoms), 1 (mild symptoms), 2 (moderate symptoms) 3 (severe symptoms) and 4 (life threatening symptoms): Did you have obstipation? Did you have involuntary bowel movements? Did you have an elevated frequency in bowel movements or diarrhea? Did you have blood loss from the rectum? Did you have difficulties in urinating? Did you have involuntary loss of urine? Did you have increased urinary urgency? Did you observe a weak urinary stream? (Appendix 3).

### Statistics

Survival was calculated by the Kaplan-Meier method using SPSS statistical software (IBM, Armonk, NY, USA). Of note, local recurrence was assessed by counting any local recurrence as event, regardless of whether this occurred as first recurrence or after metastasis.

## Results

**Fig. 2:**
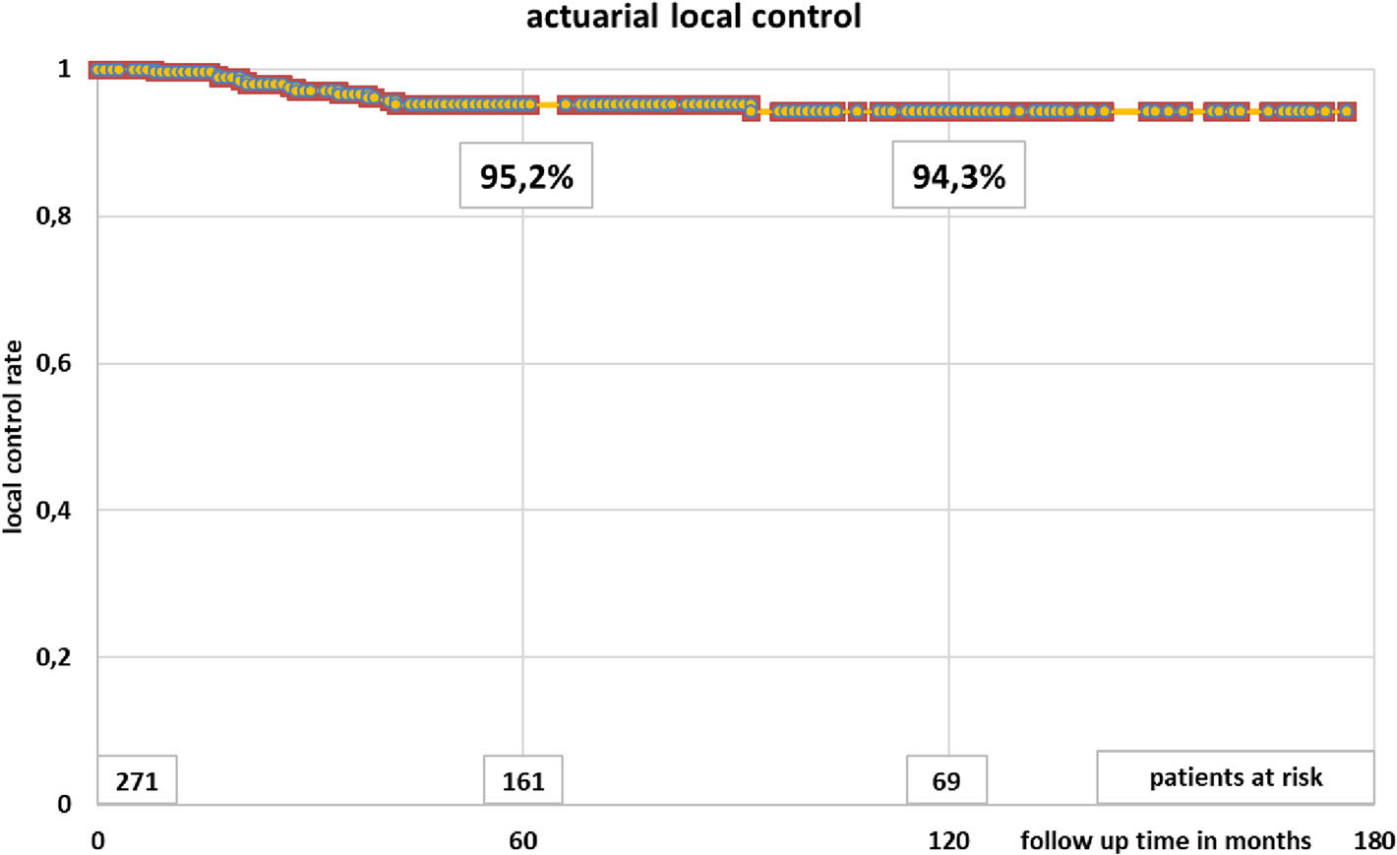
Actuarial local control after neoadjuvant short-course radiotherapy and surgery

**Fig. 3:**
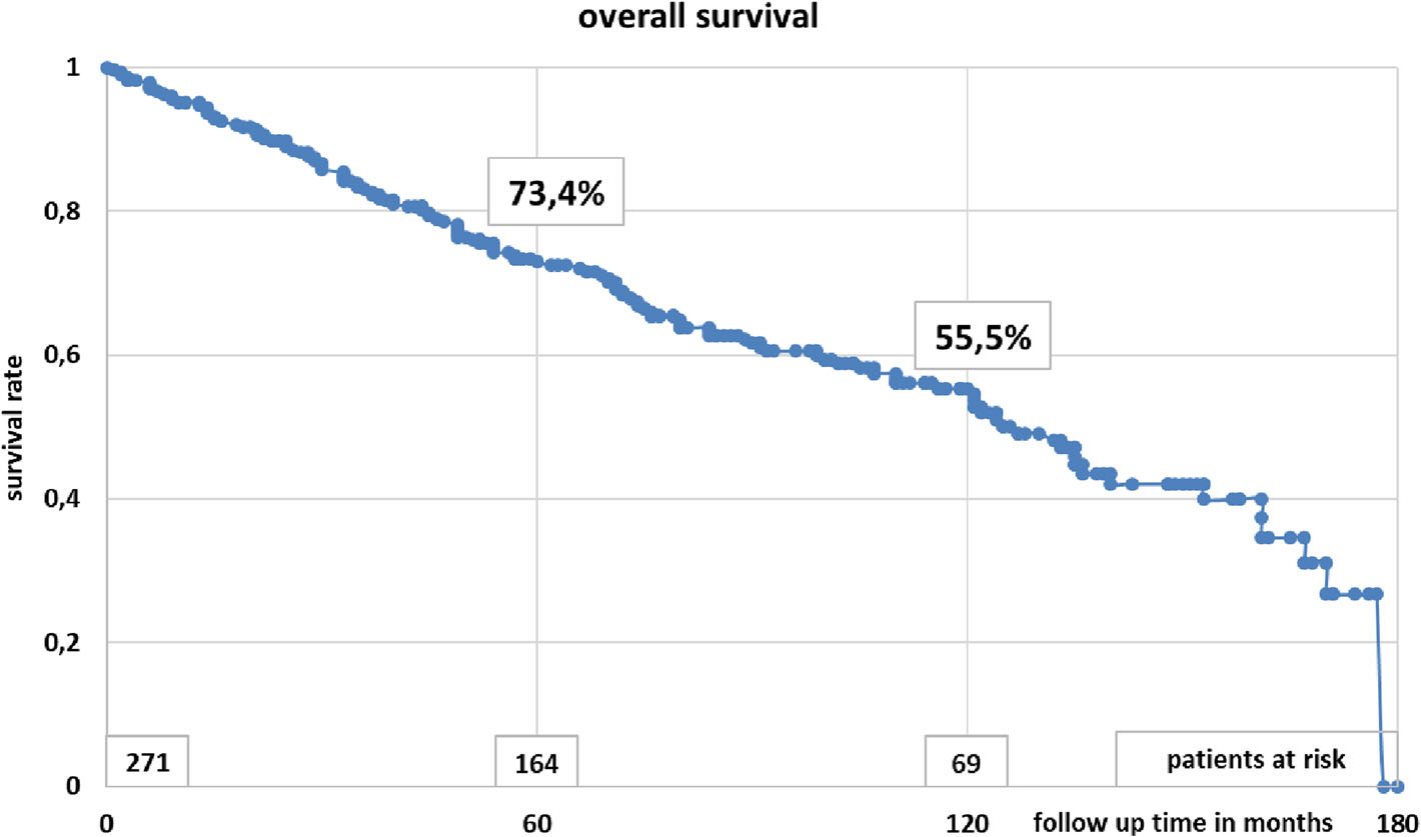
Overall survival

**Fig. 4:**
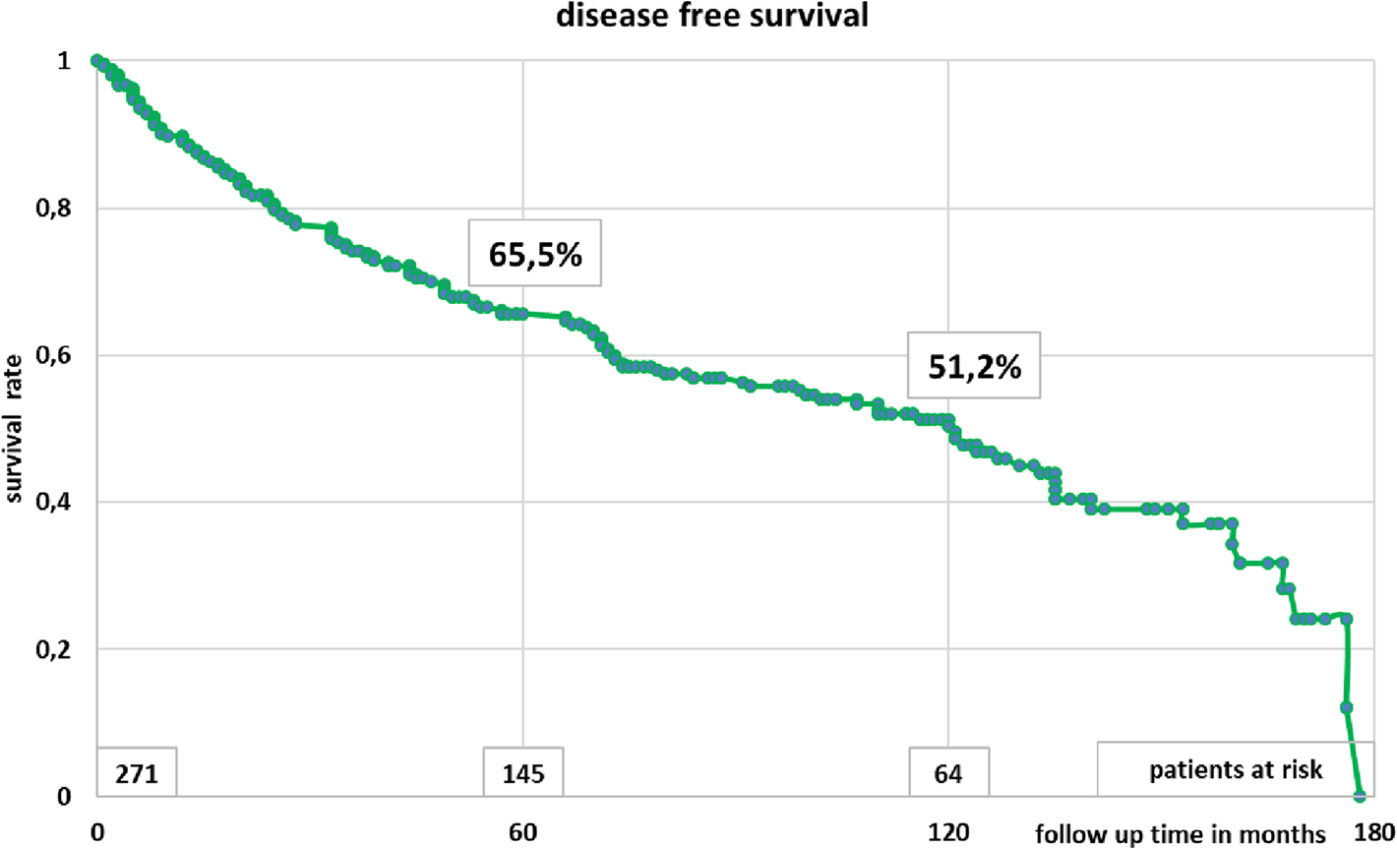
Disease-free survival

Most patients had male gender (65%) and stage II disease (65%), as shown in Table 1. All patients completed radiotherapy. Acute side effects were limited to grade 1. One hundred and thirty-seven patients (51%) were treated with an abdominoperineal resection and 134 (49%) with a low anterior resection (Table 1). Overall, 237 patients (87%) had a stoma after surgery. In 107 patients (39%) the stoma was temporary (median 179 days). Local relapse was observed in 12 patients (4.4%) after a median of 28 months (range 8–92 months), Fig. 2. Overall survival after 5 and 10 years was 73.4 and 55.5%, respectively (Fig. 3). Disease-free survival after 5 and 10 years was 65.5 and 51.2%, respectively (Fig. 4). Distant metastases occurred in 44 patients (16%) after a median interval of 29 months (range 1–92 months). Most metastases were recorded in the liver (21) or in multiple organs (15).

**Table 1:** Baseline characteristics

	All patients	Available for functional outcome	Available for functional outcome without stoma	Available for functional outcome with stoma
**Number**	271	123	66	57
**Age (median, range) in years**	70, 37–94	68, 37–94	65, 37–94	73, 50–89
**Female gender**	95 (35%)	46	25 (38%)	21 (37%)
**Male gender**	176 (65%)	77	41 (62%)	36 (63%)
**Lower third tumor**	95 (35%)	42	10 (15%)	32 (56%)
**Middle third**	149 (55%)	71	49 (74%)	22 (39%)
**Upper third**	10 (4%)	3	2 (3%)	1 (2%)
**Not documented**	17 (6%)	7	5 (8%)	2 (4%)
**Follow-up (median, range) in months**	101, 1–176	104, 13–178	114, 18–170	91, 13–176
**cTNM**				
**cT2**	15	6	4	2
**cT3**	243	115	62	53
**cT4**	8	1	0	1
**unknown**	5	1	0	1
**cN0**	97	52	24	28
**cN+**	95	40	21	19
**cNx**	79	31	21	10
**pTNM**				
**pT1**	16	7	3	4
**pT2**	87	48	27	21
**pT3**	154	60	33	27
**pT4**	12	7	3	4
**pTx**	2	1	0	1
**pN0**	155	81	41	40
**pN1**	77	31	20	11
**pN2**	37	10	4	6
**pNx**	2	1	1	0
**MRI preoperatively**				
**0**	116	43	24	19
**1**	155	80	42	38
**Abdominoperineal resection**	137	37	0	37
**Low anterior resection**	134	86	66	20

Out of 151 surviving patients, 123 (81%) provided functional outcome data after a median of 114 months post-surgery **(**Fig. 1**)**. In this subgroup, 66 patients had no stoma while 57 had a stoma. Of the patients without stoma 79% reported no LARS (0–20 points), while 9% reported LARS (21–29 points) and 12% reported serious LARS (30–42 points). The most common individual symptom was fecal urge (65% for all grades and frequencies combined), followed by increased defecation frequency (41% with > 1 per 24 h) and involuntary loss of liquid stools (39% for all frequencies combined). Among patients with stoma, 11% reported leakage of stools from the stoma bag and 17% reported unintentional release of gas/flatulence. 12% noted sore skin around the stoma, 12% reported frequent bag changes during the day and 9% during the night, 11% felt embarrassed about the stoma and 5% had problems in caring for the stoma.

Concerning urinary symptoms, 122 of 123 patients provided data and most patients reported no symptoms (grade 0) for the particular items (between 83 and 93%). In detail, the following symptom grades were reported (percent grade 0, 1, 2, 3, 4): difficulties in urinating 85, 9, 3, 2, and 0%, respectively; involuntary loss of urine 83, 3, 10, 2, and 1%, respectively; increased urinary urgency: 86, 7, 6, 0, and 1%, respectively; weak urinary stream: 93, 5, 2, 0, and 0%, respectively.

## Discussion

This study employed phone-based longitudinal assessment of functional outcome (LARS) in long-term survivors of rectal cancer managed with a multimodal approach that included neoadjuvant short-course radiotherapy, mostly in male patients with tumors in the middle third of the rectum. Median follow-up was more than 8 years. A clear majority of surviving patients (81%) provided functional outcome data. It is not possible to exclude that patients who did not provide data were dissatisfied with the outcomes and thus had more LARS symptoms and poorer quality of life than the majority of patients. Other drawbacks of the study include the following: retrospective design, lack of some baseline information that might interfere with function (comorbidity etc.), lack of information about surgical complications and several different late complications (second primary cancers, impaired sexuality etc.) and management of metastatic disease. Despite these drawbacks the present data provide interesting insights, and allow for discussion of a radiobiologically intriguing variant of the commonly employed 25 Gy short-course regimen.

Splitting the daily dose into two equally sized fractions of 2.5 Gy and adhering to a sufficiently long time interval of at least 6 h allows for reduction of late side effects such as fibrosis (reduced biologically equivalent dose for late responding normal tissues) [13], thereby reducing rectal injury [14]. The target volume and treatment planning concepts used in our study were in line with general principles [15, 16] and also included positioning on a belly board, which limits the amount of irradiated small bowel. Widder et al. used the same regimen in a smaller study (*n* = 184) with largely comparable, favorable results [9]. Their actuarial 4-year-local-recurrence rate was 2%. Disease-free survival at 4 years was 69% (65% at 5 years in our study). Postoperative mortality was 0.5% (one patient), early anastomotic leakage occurred in 11%, and anastomotic stenosis requiring treatment in 6%, of 132 patients with primary anastomosis. Randomized head to head comparisons of the 5- and 10-fraction regimen are not available. A disadvantage of the 10-fraction regimen is the fact that it is more time consuming for both patients and radiation oncology departments.

Different groups have reported results after standard 5 × 5 Gy neoadjuvant therapy. In the TME trial (1996–1999), 1530 Dutch patients with rectal cancer were randomized to TME preceded by 5 × 5 Gy or TME alone [17]. A set of questionnaires was sent to the surviving patients (*n* = 583) in 2012. The questionnaires included LARS score, EORTC QLQ-C30 and EORTC QLQ-CR29 quality of life questionnaires. The LARS score range was divided into no LARS, minor LARS, and serious LARS categories. Of the 478 respondents, 242 non-stoma patients were included in the analysis. The median interval since treatment was 14.6 years. Serious LARS was reported by 46% of all patients (56% after radiotherapy plus TME vs. 35% after TME). These figures were higher than those reported in our study population (shorter follow-up). Dutch patients with serious LARS fared worse in many quality of life domains.

The Trans-Tasman Radiation Oncology Group trial TROG 01.04 compared acute adverse events (AE) and postoperative complication rates in a randomized trial of short-course versus long-course preoperative radiotherapy [18]. Eligible patients had T3 rectal adenocarcinoma within 12 cm of the anal verge with no evidence of metastasis. Long-course therapy was 50.4 Gy administered in 28 fractions during 5.5 weeks, with infusion 5-fluorouracil, and surgery in 4 to 6 weeks. There was no 30-day operative mortality. A statistically significant higher percentage of at least one AE occurred in the long-course group (72% vs. 99%; *p* < 0.001). There were significant differences in favor of short-course therapy for grade 3 AE: radiation dermatitis (0% vs. 6%, *p* = 0.003), proctitis (0% vs. 4%; *p* = 0.016), nausea (0% vs. 3%; *p* = 0.029), fatigue (0% vs. 4%; p = 0.016) and grade 3/4 diarrhea rates (1% vs 14%; p < 0.001). No statistically significant differences in surgical complication rates were seen (53% vs. 50%; *p* = 0.68), although permanent stoma (38% vs. 30%; *p* = 0.13) and anastomotic breakdown (7% vs. 3.5%; *p* = 0.26) rates favored long-course, with perineal wound complications (38% vs. 50%; p = 0.26) in favor of short-course. Long-term outcomes were not reported in this publication.

Outcomes from Poland were based on a randomized phase 2 trial [19]. Patients (*n* = 515) with cT4 or fixed cT3 rectal cancer were randomized either to preoperative 5 × 5 Gy and three cycles of FOLFOX4 or to chemoradiation (50.4 Gy with bolus 5-FU, leucovorin and oxaliplatin). The median follow-up was 7.0 years. There was no difference in disease-free survival, hazard ratio 0.95 (95% confidence interval 0.75–1.19), at 8 years 43% vs. 41% in the short-course vs. long-course group, respectively. The rate of late complications was similar (*p* = 0.66), grade 3+ being 11% vs. 9% in the short-course vs. long-course group, respectively.

Stockholm III was a randomized phase 3 non-inferiority trial where patients with a biopsy-proven adenocarcinoma of the rectum, without signs of non-resectability or distant metastases, and planned for an abdominal resection were eligible [20]. Participants were randomly assigned to receive either 5  ×  5 Gy with surgery within 1 week or after 4–8 weeks (short-course radiotherapy with delay) or 25 fractions of 2 Gy with surgery after 4–8 weeks (long-course radiotherapy with delay). The primary endpoint was time to local recurrence. In patients with any local recurrence, median time from date of randomization to local recurrence in the pooled short-course radiotherapy comparison was 33 months (range 18–62 moths). Cumulative incidence of local recurrence in the whole trial was eight of 357 patients who received short-course radiotherapy, ten of 355 who received short-course radiotherapy with delay, and seven of 128 who received long-course radiotherapy (deemed non-inferior). Acute radiation-induced toxicity was recorded in one patient (< 1%) of 357 after short-course radiotherapy, 23 (7%) of 355 after short-course radiotherapy with delay, and six (5%) of 128 patients after long-course radiotherapy with delay. Frequency of postoperative complications was similar between all arms. However, in a pooled analysis of the two short-course radiotherapy regimens, the risk of postoperative complications was significantly lower after short-course radiotherapy with delay than after short-course radiotherapy (144 (53%) of 355 vs. 188 (41%) of 357; *p* = 0.001). Based on their findings, the authors suggested that short-course radiotherapy with delay to surgery is a useful alternative to conventional short-course radiotherapy with immediate surgery.

A Dutch study (*n* = 156) compared quality of life between short and long-course therapy from diagnosis until 24 months after treatment [21]. The patients had clinical stage T2–3 N0–2 M0 and were treated between 2013 and 2017. The EORTC-C30 and EORTC QLQ-CR29 quality of life questionnaires were employed before the start of neoadjuvant treatment (baseline) and at 3, 6, 12, 18 and 24 months after. The long-course group reported poorer emotional functioning at 3, 6, 12, 18 and 24 months (mean difference with short course: − 9.4, − 12.1, − 7.3, − 8.0 and − 7.9, respectively), and poorer global health, physical-, role-, social- and cognitive functioning at 6 months. Besides emotional functioning, all domains were comparable at 12, 18 and 24 months. Within-group changes showed a significant improvement of emotional functioning after baseline in the short-course group. Thus, long-course therapy may induce a stronger decline in short-term quality of life than short-course treatment.

In a different study, all individuals 12 to 36 months after receiving a diagnosis of colorectal cancer in England were sent a survey in January 2013 [22]. The survey responses were linked with cancer registration, hospital admissions, and radiation therapy data. Outcome measures were cancer specific (Functional Assessment of Cancer Therapy and Social Difficulties Inventory items related to fecal incontinence, urinary incontinence, and sexual difficulties) and generic (EuroQol EQ-5D questionnaire). Surveys were returned by 6713 (64%) of 10,452 patients with rectal cancer. Of these, 3998 patients were in remission after a major resection and formed the final analysis sample. Compared with those who had surgery alone, patients who received preoperative radiation therapy had higher odds of reporting poor bowel control (44% vs. 33%; odds ratio (OR) = 1.55; 95% confidence interval, range 1.26–1.91), severe urinary leakage (7% vs. 3.5%; OR = 1.69; 95% confidence interval, range 1.18–2.43), and severe sexual difficulties (34% vs. 18%; OR = 1.73; 95% confidence interval, range 1.43–2.11). Patients who received long-course chemoradiation reported significantly better bowel control than those who had short-course radiation therapy, with no difference for other outcomes. Respondents with a stoma present reported significantly higher levels of severe sexual difficulties and worse health-related quality of life than those who had never had a stoma or had undergone stoma reversal.

Overall, these studies suggest that strategies attempting toxicity reduction and quality of life improvements still are warranted, including gentle ways of radiotherapy delivery. Other current developments include use of MRI-guided radiotherapy [23], selected chemoradiation, just in cases with margin threatening tumors [24, 25], total neoadjuvant therapy [26, 27], local excision or watch and wait approaches [28], and attention towards lateral local recurrence in low rectal cancer after neoadjuvant therapy [29], e.g. in cases with persistently enlarged nodes in the internal iliac compartment.

If the results of this study with regard to local tumour control could be transferred to a more selected group of high-risk patients (deep invasion beyond the muscularis propria, threatened margin, or EMVI) remains to be determined. In any case one would expect more local failures in such a study population. However, if 10 × 2.5 Gy in five days or the commonly used short course regimen with 5 × 5 Gy or long course radio-chemotherapy would be more effective in such a high-risk population needs further (prospective) data from series with quality assured preoperative MRI-based selection criteria.

Concerning the choice of pre-operative radiotherapy in the presence of high-risk features, we proceed analogue to the ESMO guideline recommendation, e.g neoadjuvant short course radiotherapy or long course radio-chemotherapy for “bad” tumours, and long course radio-chemotherapy for “advanced/ugly” tumours or in case tumour shrinkage is needed [30]. For the short course radiotherapy regimen, our institution applies 10 × 2.5 Gy instead of 5 × 5 Gy.

## Conclusion

The present radiotherapy regimen was feasible and resulted in low rates of local relapse. Most patients reported good functional outcomes, compared for example to the older Dutch TME trial [17].

## Supplementary information

**Additional file 1.** LARS-Score [11]

**Additional file 2.** Questionnaire for patients with permanent stoma (based on EORTC QLQ-CR29)

**Additional file 3.** Bowel and urinary symptom questionnaire (based on CTCAE)

## Data Availability

The raw datasets used and analysed during the current study are not publicly available due to data privacy acts, but further general information about the project is available from the corresponding author on reasonable request.

## Abbreviations

AE: Adverse Events
APR: Abdominoperineal Resection
CT: Computed Tomography
CEA: Carcino-Embryonic Antigen
EMVI: Extramural Vascular Invasion
EORTC: European Organisation for Research and Treatment of Cancer
EORTC QLQ-CR29: EORTC Quality of Life Questionnaire (Colorectal Module containing 29 questions)
EORTC QLQ-C30: Generic EORTC Quality of Life Questionnaire for Cancer containing 30 questions
EuroQol EQ-5D: A Measure of Health Status from the EuroQol Group
ICRU: International Commission on Radiation Units and Measurements
LAR: Low Anterior Resection
LARC: Locally Advanced Rectal Cancer
LARS: Low Anterior Resection Syndrome
MRI: Magnetic Resonance Imaging
OR: Odds Ratio
TME: Total Mesorectal Excision
