# Supplementary figures and images for: Altered fractionation short-course radiotherapy for stage II-III rectal cancer: a retrospective study
Source: Radiat Oncol. 2020 May 14;15:111. doi: 10.1186/s13014-020-01566-8 (PMC7227338; doi:10.1186/s13014-020-01566-8)

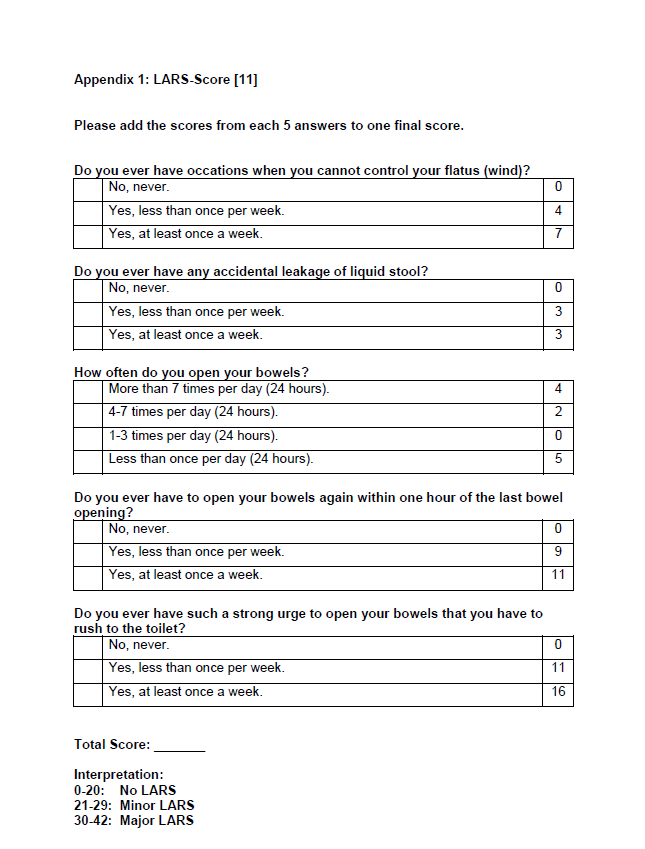

Supplement: Supplementary file 1 — Additional file 1. LARS-Score [11] [file 13014_2020_1566_MOESM1_ESM.docx]

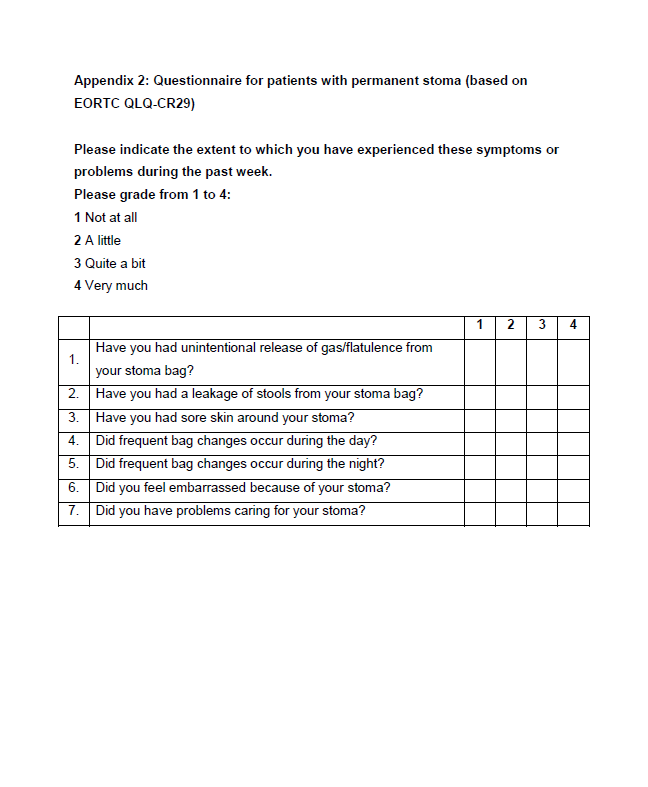

Supplement: Supplementary file 2 — Additional file 2. Questionnaire for patients with permanent stoma (based on EORTC QLQ-CR29) [file 13014_2020_1566_MOESM2_ESM.docx]

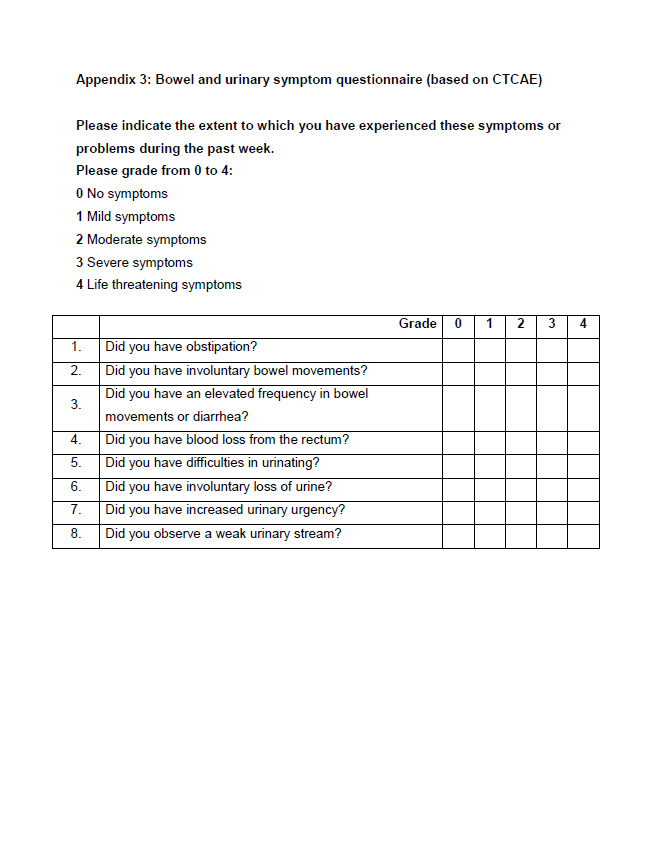

Supplement: Supplementary file 3 — Additional file 3. Bowel and urinary symptom questionnaire (based on CTCAE) [file 13014_2020_1566_MOESM3_ESM.docx]
